# Baseline liver steatosis has no impact on liver metastases and overall survival in rectal cancer patients

**DOI:** 10.1186/s12885-021-07980-9

**Published:** 2021-03-09

**Authors:** Giulia Besutti, Angela Damato, Francesco Venturelli, Candida Bonelli, Massimo Vicentini, Filippo Monelli, Pamela Mancuso, Guido Ligabue, Pierpaolo Pattacini, Carmine Pinto, Paolo Giorgi Rossi

**Affiliations:** 1 Clinical and Experimental Medicine PhD program, University of Modena and Reggio Emilia, Modena, Italy; 2 Radiology Unit, Department of Diagnostic Imaging and Laboratory Medicine, AUSL-IRCCS di Reggio Emilia, Reggio Emilia, Italy; 3 Medical Oncology Unit, AUSL-IRCCS of Reggio Emilia, Viale Risorgimento 80, 42123 Reggio Emilia, Italy; 4 Department of Medical Biotechnologies, University of Siena, Strada delle Scotte 4, 53100 Siena, Italy; 5 Epidemiology Unit, AUSL-IRCCS of Reggio Emilia, Via Amendola 2, 42122 Reggio Emilia, Italy; 6 Department of Radiology, Azienda Ospedaliero-Universitaria Policlinico di Modena, University of Modena and Reggio Emilia, 41124 Modena, Italy

**Keywords:** Liver metastases, Rectal Cancer, Liver Steatosis, Chemotherapy induced liver injury

## Abstract

**Background:**

The liver is one of the most frequent sites of metastases in rectal cancer. This study aimed to evaluate how the development of synchronous or metachronous liver metastasis and overall survival are impacted by baseline liver steatosis and chemotherapy-induced liver damage in rectal cancer patients.

**Methods:**

Patients diagnosed with stage II to IV rectal cancer between 2010 and 2016 in our province with suitable baseline CT scan were included. Data on cancer diagnosis, staging, therapy, outcomes and liver function were collected. CT scans were retrospectively reviewed to assess baseline steatosis (liver density < 48 HU and/or liver-to-spleen ratio < 1.1). Among patients without baseline steatosis and treated with neoadjuvant chemotherapy, chemotherapy-induced liver damage was defined as steatosis appearance, ≥ 10% liver volume increase, or significant increase in liver function tests.

**Results:**

We included 283 stage II to IV rectal cancer patients with suitable CT scan (41% females; mean age 68 ± 14 years). Steatosis was present at baseline in 90 (31.8%) patients, synchronous liver metastasis in 42 (15%) patients and metachronous liver metastasis in 26 (11%); 152 (54%) deaths were registered. The prevalence of synchronous liver metastasis was higher in patients with steatosis (19% vs 13%), while the incidence of metachronous liver metastasis was similar. After correcting for age, sex, stage, and year of diagnosis, steatosis was not associated with metachronous liver metastasis nor with overall survival. In a small analysis of 63 patients without baseline steatosis and treated with neoadjuvant chemotherapy, chemotherapy-induced liver damage was associated with higher incidence of metachronous liver metastasis and worse survival, results which need to be confirmed by larger studies.

**Conclusions:**

Our data suggest that rectal cancer patients with steatosis had a similar occurrence of metastases during follow-up, even if the burden of liver metastases at diagnosis was slightly higher, compatible with chance.

**Supplementary Information:**

The online version contains supplementary material available at 10.1186/s12885-021-07980-9.

## Background

The estimated global prevalence of liver steatosis is around 25% and projected to be 33.5% in 2030 [1]. Since non-alcoholic fatty liver disease and metabolic syndrome are associated with increased incidence of colorectal cancer (CRC) [2, 3], the expected prevalence of liver steatosis in CRC patients is at least similar or possibly higher than that estimated in the general population. Moreover, cancer patients have a strong risk of developing liver steatosis and steatohepatitis as a consequence of anticancer therapies, especially chemotherapy drugs such as 5-Fluorouracil, an anti-metabolite (anti-pyrimidine), and irinotecan, a cytotoxic anti-tumour molecule of the DNA topoisomerase inhibitor class. This liver side effect is particularly described in patients with metastatic CRC [4, 5], who develop steatosis in 30–47% cases and steatohepatitis in about 20% of those cases treated with irinotecan [6, 7].

CRC is the third most incident cancer worldwide in western countries [8]; rectal cancer accounts for about 27% of the total colorectal cancer incidence in Italy [9]. The most frequent sites for metastases in rectal cancer are liver and lung (12.3 and 5.6%, respectively) [10]. In the first decade of 2000, during the implementation of screening programmes in Italy, the proportion of patients presenting with synchronous distant metastases was 16% [9]. Despite the improvement in locally-advanced rectal cancer treatment, including neoadjuvant therapy and standardized surgical treatment, distant metastases after curative-intent treatment are still common (25–40% in the first 5 years), with lung and liver as the most common sites [11–13]. Liver recurrence is one of the most important prognostic factors for rectal cancer patients, influencing survival more than does the occurrence of lung metastases [13]. Personalized biomarkers to correctly stratify the risk of liver recurrence could be useful to improving the management of those patients.

Several studies have investigated the relationship between liver steatosis and liver metastasis occurrence in patients with solid tumours. Microenvironment changes induced by steatosis, such as inflammation and stellate cells activation, may influence neoangiogenesis [14–16] and may either favour or interfere with the process of metastasizing in the liver. In a study of CRC patients who underwent liver resection for synchronous metastases, steatosis was an independent risk factor for liver recurrence [17], while in another study of CRC patients without baseline liver metastases, steatosis was associated with a lower incidence of liver metastases during follow-up [18]. Discordant results on this topic have also been reported for other primitive cancer sites, including breast cancer and non-small cell lung cancer [19, 20].

Studying the complex association between liver steatosis and metastases is even more complicated if we take into account two possible diverging biases induced by steatosis: 1) the masking effect of liver steatosis on the detection of liver metastases, i.e., hypovascular metastases, may be difficult to detect by computed tomography (CT) scan in a fatty liver [21]; 2) hepatic diffuse disease may lead to performing more and different imaging tests, which can increase the probability of detecting liver metastases and thus classifying them as synchronous instead of metachronous. Our hypothesis is that these biases may be responsible for the discordant results found in previous studies.

The aim of this study was to determine whether baseline imaging-defined liver steatosis is a risk factor for liver metastasis occurrence in patients with stages II-IV rectal cancer. We also aimed at evaluating the impact of the presence of baseline liver steatosis on overall survival. Finally, in patients with locally advanced rectal cancer, we evaluated the association between chemotherapy-induced liver damage and liver metastasis occurrence and overall survival after the end of chemotherapy.

## Methods

### Study design and population

In this retrospective observational study, all consecutive patients diagnosed with stage II-IV rectal cancer between 2010 and 2016 in the province of Reggio Emilia, with available CT scan performed at the time of diagnosis for staging purposes, were included. Patients with baseline CT not suitable for evaluation of presence/absence of liver steatosis, including unavailability of liver unenhanced CT images or diffuse liver parenchymal derangement, were excluded.

The rationale of including both locally advanced and metastatic patients (stages II-IV) derives from the possible masking effect of liver steatosis on liver metastasis detection. Since liver steatosis may affect the sensitivity of CT scan in the detection of liver metastases, patients with and without liver steatosis may have a different probability of having undetected metastases at baseline, with a consequent possible excess of liver metastasis incidence in the group with undetected metastases. By excluding stage IV patients, we would have introduced a bias in the comparison of cumulative incidence of liver metastases in patients with and without steatosis. This bias is overcome by the comparison of both liver metastasis prevalence at baseline (including stage IV patients) and cumulative incidence during follow-up.

### Clinical data

Data on patient health status, rectal cancer diagnosis, staging, therapy and outcomes and liver function tests were extracted from the local population-based Cancer Registry and from the electronic medical records of all the Local Health Authority hospitals of the province, which report all inpatient and outpatients procedures. The Reggio Emilia Cancer Registry includes all malignant cancer cases diagnosed in the Reggio Emilia province since January 1, 1996. Cancer site and morphology are coded according to International Classification of Diseases for Oncology – 3rd Edition (ICD-O-3). Clinical TNM staging for rectal cancer in our centre is based on magnetic resonance (MR) imaging and endorectal ultrasonography (US) for local staging and on CT scan for evaluation of distant metastases, followed by other exams such as contrast-enhanced US, MR or positron emission tomography (PET) if needed. CT images were retrieved from the radiology information system - picture archiving and communication system (RIS-PACS) of the Local Health Authority.

### CT scan evaluation of liver steatosis

CT scans performed at the time of diagnosis were retrospectively reviewed by a single radiologist with 7 years of experience in liver imaging, blinded to clinical data and patient outcomes. Mean liver and spleen attenuation values (Hounsfield units, HU) were obtained by placing eight regions of interest (ROIs) in the liver and three ROIs in the spleen [22], being careful to exclude vessels, bile ducts, focal lesions, focal fatty changes or focal fatty sparing and visceral margins. Steatosis was defined as present for absolute liver density < 48 HU and/ or liver-to-spleen ratio < 1.1. A moderate-to-severe degree of steatosis was defined for absolute liver density < 40 HU and/ or liver-to-spleen ratio <  0.8 and/ or liver-spleen difference < − 10 HU [23].

### Outcome measures

Liver metastases already present at the time of diagnosis and those occurring during a follow-up period of at least 2 years were considered separately. Follow-up duration for the occurrence of metastases was considered from disease diagnosis to the last imaging exam, excluding the presence of metastases. Overall survival (OS) was defined as the time from disease diagnosis to all-cause death or the last clinical evaluation.

### Subgroup analysis on post-chemotherapy liver damage

The effect of chemotherapy-induced liver damage was evaluated in a subgroup of patients with locally advanced rectal cancer treated with neoadjuvant chemotherapy and with an available CT scan performed within 4 months from the cessation of chemotherapy, without baseline liver steatosis. At our institution, the standard neoadjuvant chemotherapy treatment for locally advanced rectal cancer patients includes fluorouracil and oxaliplatin, while irinotecan is rarely used.

By comparing baseline and post-neoadjuvant chemotherapy CT scans and liver function tests, liver damage was defined as at least one of the following: 1) appearance of liver steatosis, 2) liver volume change of ≥10%, 3) increase in liver function tests (doubled in values and AST > 40 U/L; ALT > 49 U/L; GGT > 73 U/L).

In this subgroup of patients, baseline and post-neoadjuvant chemotherapy liver volume was retrospectively assessed by a blinded dedicated post-processing technologist supervised by a blinded abdominal radiologist through liver manual segmentation on portal venous phase CT scans.

Patients with baseline liver steatosis were excluded from this analysis since it was impossible to apply one of the three criteria defining damage in those patients.

Follow-up was defined as starting from 4 months after neoadjuvant chemotherapy end date up to event or last imaging or oncologic evaluation for metastasis occurrence, or to death or last update of mortality registry (August 31, 2019) for overall survival.

### Ethics

This observational retrospective study was performed in accordance with the Declaration of Helsinki and was approved by the local ethics committee (“Comitato Etico dell’Area Vasta Emilia Nord”) with protocol number 2019/0079373. The need for informed consent was waived due to the retrospective nature of the study.

Nevertheless, the investigators asked all patients presenting to any of the participating institutions for any clinical reasons for consent.

### Statistical analyses

We present the distribution of patient characteristics, therapies received and completeness of follow-up by stage at diagnosis. Cumulative incidence of metastasis is presented in graphs using Nelson-Aalen cumulative hazard function.

The association between steatosis and metastasis at baseline was assessed with a logistic model and reported as odds ratio with relative 95% confidence intervals (95% CI). All cases were included in the model. The association between steatosis and metastasis detection during follow-up was assessed with Cox proportional hazards regression model and reported as hazard ratios with relative 95% CI. Only cases free from liver metastases at baseline were included in the model. Models were adjusted for variables identified a priori: age (as continuous variable), sex, calendar period (i.e. year of diagnosis), and stage. We chose not to adjust for risk factors that may lie on the same causal chain linking liver steatosis and liver metastases.

Median OS was computed by using the reverse Kaplan Meier method. Cox models for OS were constructed as those for liver metastases, but in this case, we performed two analyses, one including and the other excluding patients with liver metastases at baseline.

The association between post-chemotherapy liver damage and incidence of metastases was assessed with an exact logistic model [24]. The model included only cases that were free from steatosis at baseline, had completed chemotherapy, and were free of metastases at the first post-treatment imaging assessment (i.e. a CT performed between February 23, 2011 and May 4, 2017). To select the variables to include in final models, we built a standard logistic model including age, sex, calendar period, and stage: variables that were not associated with the outcome were excluded (*p* > 0.5), while the remaining variables were included in the exact logistic model. Odds ratios (ORs) were compared with hazard ratios (HRs) obtained with similar Cox models in order to exclude that differences in length of follow-up influenced the results.

We did not perform any formal statistical tests; *p*-values as well as 95% confidence intervals should be interpreted as an indication of the probability that the observed differences occurred under the null hypothesis without a pre-fixed threshold of significance.

## Results

### Study population

**Fig. 1:**
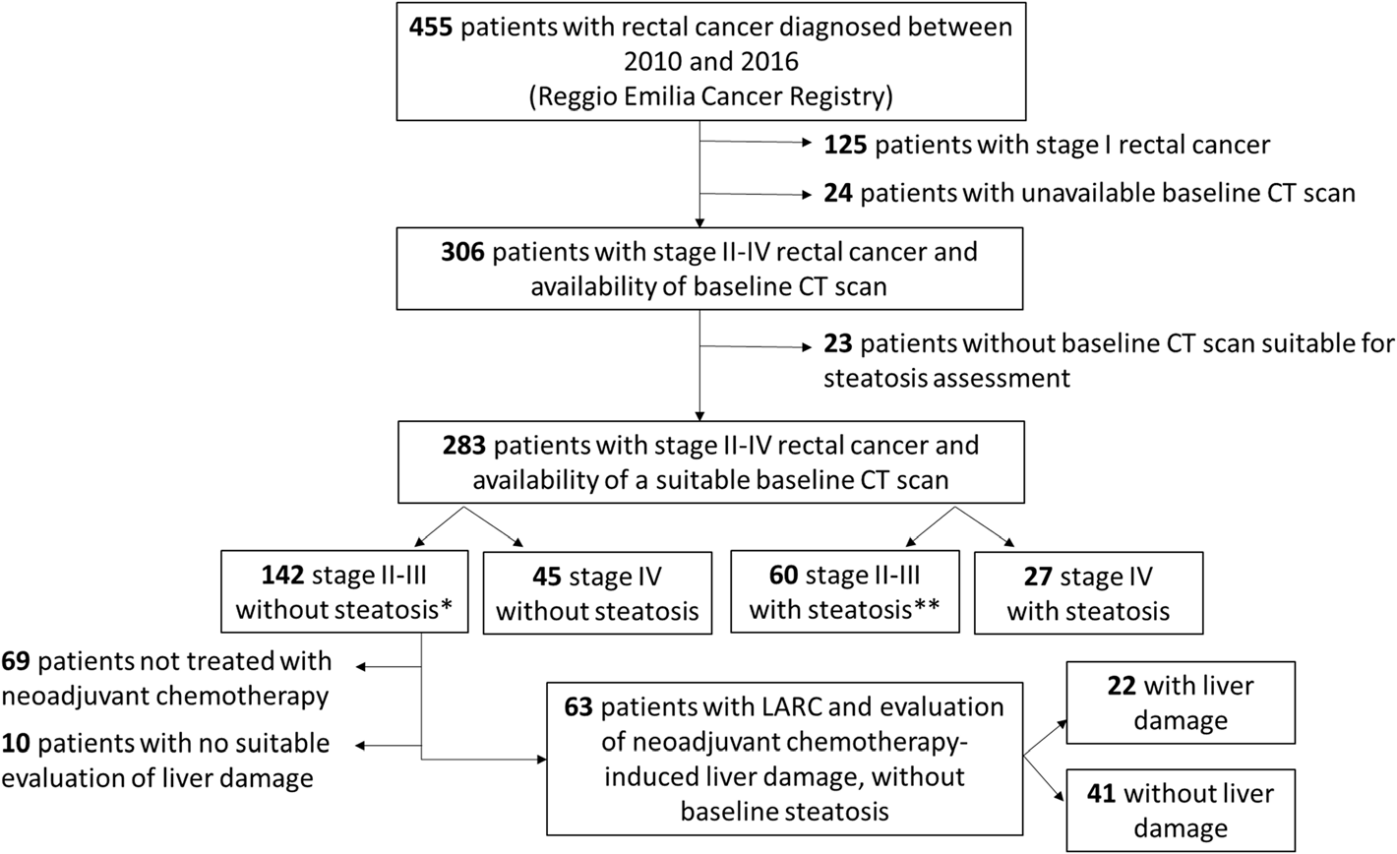
Patient flowchart for the main study and subgroup analysis. * + 6 patients stage XM0; ** + 3 patients stage XM0. TNM stage refers to clinical staging. LARC (locally advanced rectal cancer)

From 2010 to 2016, 465 patients were diagnosed with rectal cancer in the Reggio Emilia province. After excluding patients with stage I rectal cancer and patients with unavailable or unsuitable CT scan at diagnosis, 283 patients were included in the main analysis (Fig. 1).

Of these 283 patients, 116 (41%) were females. Mean age was 68 ± 14 years. Disease stage at diagnosis was II in 77 (27%) patients, III in 125 (44%) patients and IV in 72 (25%) patients. For 9 patients the stage was unknown but distant metastases were excluded (stage XM0). Patient characteristics in different disease stages are reported in Supplementary Table S1, Additional file.

Liver steatosis was present at baseline in 90 (31.8%) patients, of whom 19 (21.1%) had severe steatosis Table 1). Baseline liver metastases were present in 42 (15%) patients, while 26 (11%) patients developed liver metastases during follow-up. Median follow-up duration for liver metastasis detection was 31 months. Median follow-up duration for overall survival was 45 months. The overall number of deaths occurred during follow-up was 152 (54%) and among them 27 (18%) were for causes different from CRC. Baseline characteristics in patients with and without steatosis are reported in Table 2, while follow-up and treatment characteristics are listed in Supplementary Table S2, Additional file, and baseline characteristics in patients with moderate/severe steatosis are reported in Supplementary Table S3, Additional file.

**Table 1:** Baseline liver characteristics including CT indices of steatosis and liver function tests

	No Liver Steatosis (***n*** = 193; 68.2%)	Mild liver steatosis (***n*** = 71; 25.1%)	Moderate/Severe liver steatosis (***n*** = 19; 6.7%)
Liver density (HU); mean (SD)	58.35 (5.98)	49.38 (5.31)	34.84 (10.20)
Liver-to-spleen density ratio; median (range)	1.25 (1.09; 2.1)	1.09 (0.89;1.47)	0.78 (0.21; 1.03)
Liver-spleen density difference (HU); median (range)	12 (5; 33)	3.54 (−6; 15)	−10.53 (−38; 1)
AST (U/L); median (range) ^a^	17 (10; 124)	25.57 (16; 48)	22.46 (7; 118)
AST > 40 U/L; n (%) ^a^	3 (2.7)	4 (6.6)	1 (7.1)
ALT (U/L); median (range) ^a^	14 (7; 94)	22.11 (7; 143)	30.29 (8; 76)
ALT > 49 U/L; n (%) ^a^	2 (1.8)	2 (3.2)	2 (14.3)
GGT (U/L); median (range) ^a^	17 (6; 280)	55.95 (7; 763)	49.21 (13; 171)
GGT > 73 U/L; n (%) ^a^	4 (4.0)	9 (16.4)	3 (21.4)

CT liver characteristics and liver function tests in patients with and without CT-defined liver steatosis. *SD* standard deviation, *HU* Hounsfield Units. ^a^ missing values were: in no liver steatosis group 83, 81, and 92 for AST, ALT, and GGT, respectively, in mild steatosis group 10, 9, and 16 for AST, ALT, and GGT, respectively, and in moderate/severe steatosis group 5 for all the three liver function tests.

**Table 2:** Baseline characteristics in patients with and without baseline CT-defined liver steatosis

		No Liver Steatosis (***n*** = 193; 68.20%)				Liver Steatosis (***n*** = 90; 31.80%)			
		Overall	Synchronous liver metastases (***n*** = 25)	Metachronous liver metastases (***n*** = 17)	Deaths (***n*** = 106)	Overall	Synchronous liver metastases (***n =*** 17)	Metachronous liver metastases (***n =*** 9)	Deaths (***n*** = 46)
Age; mean (SD)		68.27 (14.3)				66.81 (13.23)			
Sex; n (%)	Male	106 (54.9)	15	9	62	61 (67.8)	11	4	30
	Female	87 (45.1)	10	8	44	29 (32.2)	6	5	16
Stage; n (%)	II	56 (29.0)	–	5	29	21 (23.3)	–	1	3
	III	86 (44.6)	–	8	32	39 (43.3)	–	7	17
	IV	45 (23.3)	25	4	41	27 (30.0)	17	1	23
	XM0	6 (3.1)	–	–	4	3 (3.3)	–	–	3
Grade; n (%)	Well	1 (0.5)	0	0	0	2 (2.2)	1	0	2
	Moderately	64 (33.2)	4	2	25	37 (41.1)	6	4	13
	Poorly	79 (40.9)	10	11	47	29 (32.2)	3	1	16
	Missing	49 (25.4)	11	4	34	22 (24.4)	7	4	15

*TNM stage refers to clinical staging. Stage XM0 was considered for patients with unknown local staging but exclusion of distant metastases with CT scan. SD* Standard deviation

### Association between liver steatosis and liver metastases

**Fig. 2:**
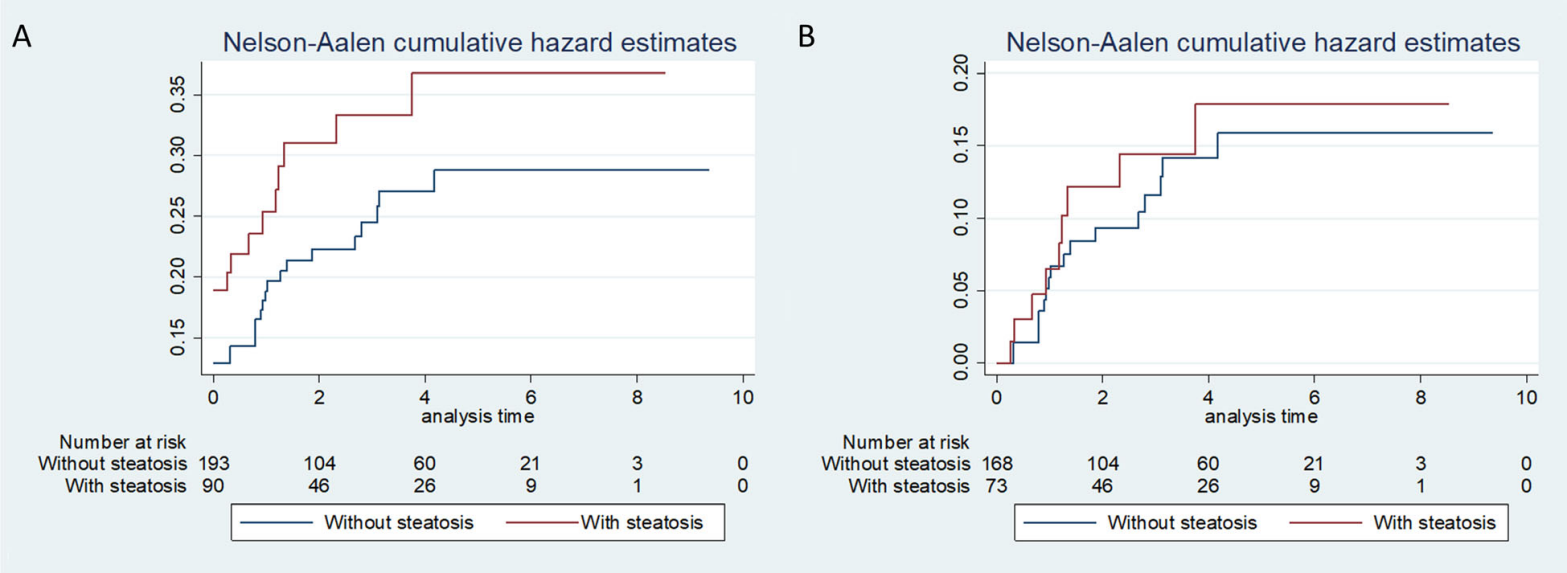
Cumulative incidence of liver metastases in all patients subdivided according to absence/presence of liver steatosis, including all metastases (**a**) and only metastases occurring during follow-up (**b**). Note that in graph A, cumulative hazard curves start from the observed values of synchronous metastases, i.e. 13.0% in patients without steatosis and 18.9% in patients with steatosis

The prevalence of liver metastases at baseline was slightly higher in patients with liver steatosis (17/90, 19%) than in those without (25/168, 13%), while the incidence of liver metastases during follow-up was similar in patients with and without liver steatosis: 9 out of 73 (12%) and 17 out of 168 (10%), respectively (Fig. 2). The final cumulative incidence of metastases was slightly higher in patients with steatosis.

After correcting for possible confounders, liver steatosis was slightly associated with the presence of liver metastases at baseline, even if the difference in prevalence may have been due to chance; the association was almost null when considering the metachronous liver metastases occurring during follow-up (Table 3).

**Table 3:** Impact of liver steatosis on patient outcomes

***Variables***	***Synchronous metastases*** (***n*** = 283 patients; ***n*** = 42 metastases)			***Metachronous metastases*** (***n*** = 241 patients; ***n*** = 26 metastases)			***Overall survival*** (***n*** = 283 patients; ***n*** = 152 deaths)			***Overall survival excluding patients with baseline liver metastases*** (***n*** = 241 patients; ***n*** = 73 deaths)		
	***OR***	***95%CI***	***p***	***HR***	***95%CI***	***p***	***HR***	***95%CI***	***p***	***HR***	***95%CI***	***P***
Liver steatosis	1.58	0.79–3.14	0.20	1.25	0.55–2.85	0.60	0.92	0.64–1.32	0.65	0.66	0.35–1.22	0.19
Age at diagnosis	1.01	0.99–1.04	0.43	0.99	0.97–1.02	0.61	1.03	1.01–1.04	< 0.001	1.05	1.03–1.07	< 0.001
Sex	0.90	0.45–1.77	0.75	1.66	0.76–3.66	0.21	1.03	0.74–1.44	0.89	0.76	0.43–1.36	0.36
Stage	–	–	–	1.78	1.00–3.20	0.05	2.15	1.76–2.62	< 0.001	2.51	1.64–3.82	< 0.001
Year of diagnosis	1.01	0.85–1.19	0.91	0.88	0.73–1.08	0.22	0.98	0.90–1.06	0.64	0.82	0.71–0.94	0.006

*Logistic multivariate models for liver metastasis presence at baseline (model includes patients with liver metastases at baseline) and Cox proportional hazards regression models for occurrence of liver metastases during follow-up (after exclusion of patients with baseline liver metastases) and for overall survival (both including all patients and after exclusion of patients with baseline liver metastases). The variable of interest was steatosis at baseline; adjusting variables (age, sex, stage, and calendar period) included in the models were selected* a priori *for their known impact on disease. TNM stage refers to clinical staging.*

### Impact of liver steatosis on overall survival

**Fig. 3:**
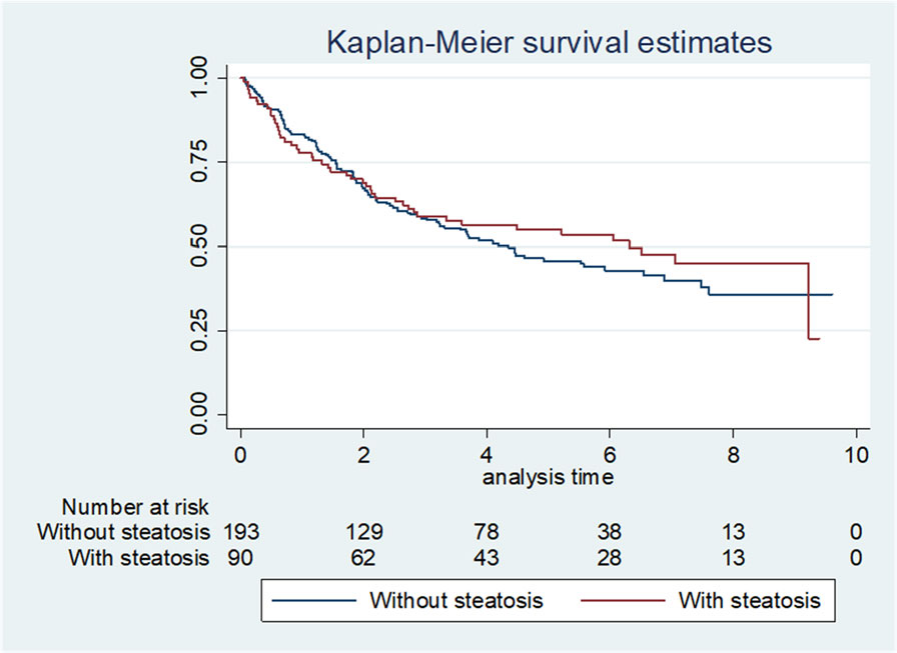
Overall survival in patients with and without baseline steatosis. All patients were included in this analysis

No difference was found in terms of overall survival between patients with and without baseline liver steatosis (*p* = 0.46) (Fig. 3). After correcting for age at diagnosis, sex, year of diagnosis and stage, liver steatosis did not affect overall survival (Table 3).

### Post-chemotherapy liver damage

Of the 202 patients with locally advanced rectal cancer, 142 had no baseline liver steatosis. Among these, patients who were not treated with neoadjuvant chemotherapy, who did not complete it, or who did not have a suitable assessment of damage (liver function tests or CT scan within 4 months from chemotherapy end date) were excluded. After also excluding patients who died (*n* = 1) or had liver metastases (*n* = 2) before the start of follow-up (i.e. 4 months after the end of neoadjuvant chemotherapy), 62 patients were included in the subgroup analysis on post-chemotherapy liver damage (Supplementary Tables S4, S5, and S6, Additional file).

Liver damage was found in 24 (38.7%) patients, 17 (70.8%) of whom had CT-defined liver steatosis appearance or liver volumetric change and 11 (45.8%) of whom had significant increase in liver function tests.

**Fig. 4:**
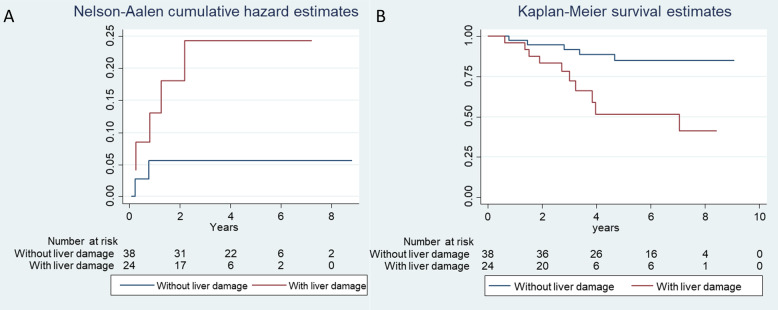
Cumulative incidence of liver metastases (**a**) and overall survival (**b**) in patients with and without post-neoadjuvant chemotherapy liver damage

In this small group of patients, those experiencing post-chemotherapy liver damage had a higher occurrence of liver metastases during follow-up, even if based only on 5 vs 2 events in the group without vs with liver damage, respectively, and worse overall survival, with 10 vs 5 events, respectively (Fig. 4). After adjusting for potential confounders, exact logistic regressions showed that the ORs of post-chemotherapy liver damage were 6.0 (95% CI 0.82 to 74.0) for liver metastases, and 5.9 (95% CI 1.37–30.9) for all-cause mortality. Point estimates were similar when using Cox proportional hazard models (HR 6.2 for liver metastases, HR 4.4 for OS).

## Discussion

In this cohort of patients with stage II-IV rectal cancer, the prevalence of CT-defined liver steatosis was 31.8%, similar or slightly higher than that reported in the general population [1]. The prevalence of steatosis was slightly higher in stage IV.

The prevalence of liver metastases at diagnosis was slightly higher in patients with steatosis (OR 1.6, 95% CI 0.8–3.0). Although the difference could be due to chance, it is interesting that the higher prevalence of steatosis in stage IV is completely justified by the excess of liver metastases present in patients with steatosis.

Our data show that steatosis is not associated with a higher probability of developing a metastasis during follow-up (HR 1.25, 95% CI 0.55–2.85). Nevertheless, cumulative incidence was higher in patients with steatosis due to the higher prevalence at diagnosis. This observation has some implications that should be considered when comparing our study with others, because some of the metastases detected at baseline in our study may have been detected during follow-up in other studies and vice versa, depending on the intensity of diagnostic imaging procedures adopted for staging. The consequence is that studies including only patients without metastases at diagnosis may show only one part of the phenomenon.

Existing studies on the effect of liver steatosis on the risk of developing liver metastases in CRC patients present ambiguous results. In fact, the results from studies on patients who underwent primary CRC resection [18, 25, 26] suggest a protective role of steatosis in terms of both synchronous and metachronous liver metastasis. On the other hand, studies on patients in follow-up after resection of liver metastases [17, 27] show an equal or higher risk of developing a new metastasis in those with steatosis. The main differences between our study and the existing literature are the inclusion of all stage II-IV rectal cancers and the focus exclusively on rectal cancer, which is a biologically distinct entity compared to colon cancer, with different characteristics also in terms of the probability of liver metastases [10, 13]. Moreover, while some other studies used CT for the assessment of liver steatosis [17], others adopted pathological examination on liver biopsy specimens [25, 28]. Although the use of CT for the assessment of liver steatosis has been validated [29], the comparability between studies which adopted different techniques may be affected.

Based on our results, it seems that both the biases that we hypothesized when designing this study were not confirmed. A masking effect of liver steatosis on the detection of metastases would have resulted in a lower prevalence of metastases at baseline, followed by a liver metastasis incidence excess in patients with steatosis. On the other hand, the increased detection of metastases due to more and different imaging tests in patients with steatosis would have induced an anticipation in liver metastasis diagnosis, resulting in a higher prevalence of baseline metastasis followed by a reduced occurrence during follow-up in patients with steatosis. Our results do not reflect any of these conditions. As our curves of metastasis occurrence after diagnosis were perfectly parallel in the two groups, if we had had undetected metastases, these would be similar in the two groups. Consequently, either no masking effect or detection bias was present or the two compensated each other perfectly.

We observed lower overall survival and a higher risk of liver metastases in patients with post-chemotherapy liver damage. Due to the small sample size, our results are very imprecise and could be due to chance. A previous study reported an association between chemotherapy-induced liver damage and improved survival after CRC liver metastasis resection [30]. The discrepancy between these two results, despite the different contexts and study designs, should stimulate the conception of larger studies on this topic. Further studies should be designed to confirm such association with larger numbers, but also to understand what the direction of causality is. In fact, as most of the liver metastases in patients with liver damage in our study were diagnosed a few months after the end of chemotherapy, we cannot exclude that liver damage may be a sign of existing micrometastases [31] and not a risk factor for developing a metastasis. In other words, the liver changes that we classified as chemotherapy-induced liver damage may represent generic liver characteristics (increase in liver function tests and liver volume and decreased liver density) linked to liver metastases, as a sort of early biomarker of undetectable metastasization. This reverse causality interpretation could also explain the discrepancies with previous studies on patients surviving after liver metastasis resection.

This study has several limitations. Firstly, it was conducted retrospectively. Nevertheless, starting from a population-based registry allowed us to understand whether and how some of the biases of a retrospective cohort study could occur. The study flowchart shows that very few cases were excluded due to the lack of adequate imaging. We cannot be certain that procedures for assessing metastases were conducted in the same way in all patients, and differences could be linked to liver conditions. Secondly, our sample size was small, particularly for the secondary aim of assessing liver damage as a risk factor. These were pre-planned analyses and therefore reported results are not driven by data, meaning that the reported *p*-values can be interpreted as true test of hypothesis; we therefore decided not to define a threshold to reject the hypothesis, but only to present a confidence interval to reflect on the possible implication of this association. Data on factors associated with liver steatosis, such as BMI, presence of metabolic syndrome, viral infections, or alcohol intake, were available only for few patients. However, by adjusting for these factors, which may lie on the same causal chain linking steatosis with liver metastases, there would be a high chance of hiding the effect of the more distal risk factors in favour of the proximal ones. Finally, steatosis was assessed by CT scan, which is known to be highly specific but presents low sensitivity, especially for mild steatosis [23], and not by MR or histological examination, which are more precise. However, CT scan was the only test available for the vast majority of rectal cancer patients at baseline.

## Conclusion

Our data suggest that rectal cancer patients with and without steatosis have a similar occurrence of metastases during follow-up, despite our observing a slightly higher burden of liver metastases at diagnosis in patients with steatosis, compatible with chance. In this preliminary analysis, liver damage after chemotherapy was associated with a higher occurrence of liver metastases; this association and its direction should be further assessed by larger prospective studies.

## Supplementary Information

**Additional file 1: Supplementary Table S1** Clinical data, CT-defined liver steatosis, and outcomes (metastases, deaths) in patients subdivided according to stage**. Supplementary Table S2** Follow-up and treatment characteristics in patients with and without liver steatosis. **Supplementary Table S3** Baseline characteristics of patients with moderate/severe steatosis. **Supplementary Table S4** CT characteristics and liver function test changes after neoadjuvant chemotherapy, defining the presence or absence of post-chemotherapy liver damage. **Supplementary Table S5** Follow-up, treatment, and outcome measures in patients with and without post-chemotherapy liver damage. **Supplementary Table S6** Demographic and clinical characteristics of patients with and without post-chemotherapy-induced liver damage, reported also in patients with events.

## Data Availability

The datasets generated and/or analysed during the current study are not publicly available due to privacy and ethical restrictions but are available from the corresponding author on reasonable request.

## Abbreviations

CI: Confidence Interval
CRC: Colorectal Cancer
CT: Computed Tomography
LM: Liver Metastasis
OS: Overall Survival
RC: Rectal Cancer
HU: Hounsfield Unit
ROI: Region of interest
