# Supplementary material for: Baseline liver steatosis has no impact on liver metastases and overall survival in rectal cancer patients
Source: BMC Cancer. 2021 Mar 9;21:253. doi: 10.1186/s12885-021-07980-9 (PMC7941741; doi:10.1186/s12885-021-07980-9)
Supplement: Supplementary file 1 — Additional file 1: Supplementary Table S1 Clinical data, CT-defined liver steatosis, and outcomes (metastases, deaths) in patients subdivided according to stage. Supplementary Table S2 Follow-up and treatment characteristics in patients with and without liver steatosis. Supplementary Table S3 Baseline characteristics of patients with moderate/severe steatosis. Supplementary Table S4 CT characteristics and liver function test changes after neoadjuvant chemotherapy, defining the presence or absence of post-chemotherapy liver damage. Supplementary Table S5 Follow-up, treatment, and outcome measures in patients with and without post-chemotherapy liver damage. Supplementary Table S6 Demographic and clinical characteristics of patients with and without post-chemotherapy-induced liver damage, reported also in patients with events. [file 12885_2021_7980_MOESM1_ESM.docx]

Title:

Baseline liver steatosis has no impact on liver metastases and overall survival in rectal cancer patients

Authors: Besutti Giulia^1,2^, Damato Angela^3,4^, Venturelli Francesco^5^, Bonelli Candida^3^, Vicentini Massimo^5^, Monelli Filippo^1,2^, Mancuso Pamela^5^, Ligabue Guido^6^, Pattacini Pierpaolo^2^, Pinto Carmine^3^, Giorgi Rossi Paolo^5^.

Affiliations:

1 Clinical and Experimental Medicine PhD program, University of Modena and Reggio Emilia, Modena, Italy.

2 Radiology Unit, Department of Diagnostic Imaging and Laboratory Medicine, AUSL-IRCCS di Reggio Emilia, Reggio Emilia, Italy.

3 Medical Oncology Unit, AUSL-IRCCS of Reggio Emilia, Viale Risorgimento 80, 42123 Reggio Emilia, Italy.

4 Department of Medical Biotechnologies, University of Siena, Strada delle Scotte 4, 53100 Siena, Italy.

5 Epidemiology Unit, AUSL-IRCCS of Reggio Emilia, Via Amendola 2, 42122 Reggio Emilia, Italy.

6 Department of Radiology, Azienda Ospedaliero-Universitaria di Modena, University of Modena and Reggio Emilia, 41124, Modena, Italy.

Corresponding Author:

Filippo Monelli, MD

Clinical and Experimental Medicine PhD program, University of Modena and Reggio Emilia, Modena, Italy; Radiology Unit, Department of Diagnostic Imaging and Laboratory Medicine, AUSL-IRCCS di Reggio Emilia, Reggio Emilia, Italy. Email: mofilippo@hotmail.it.

Supplementary Table S1: Clinical data, CT-defined liver steatosis, and outcomes (metastases, deaths) in patients subdivided according to stage

| **Table S1** | | **Stage II (n=77)** | **Stage III (n=125)** | **Stage IV (n=72)** | **Stage XM0 (n= 9)** |
| --- | --- | --- | --- | --- | --- |
| Age; mean (SD) | | 70.56 y (13.22) | 65.40 y (14.07) | 67.51 y (13.94) | 80.00 y (8.57) |
| Sex; n (%) | Male | 49 (63.64%) | 68 (54.40%) | 44 (61.11%) | 6 (66.67%) |
|  | Female | 28 (36.36%) | 57 (45.60%) | 28 (38.89%) | 3 (33.33%) |
| Median follow-up (IQR) for Overall Survival | | 56 (32-76) | 53 (30-78) | 16 (7-32.5) | 7 (1-39) |
| T Stage;  n (%) | 1 | 0 (0.00%) | 3 (2.40%) | 0 (0.00%) | 0 (0.00%) |
|  | 2 | 0 (0.00%) | 16 (12.80%) | 0 (0.00%) | 0 (0.00%) |
|  | 3 | 65 (84.42%) | 80 (64.00%) | 0 (0.00%) | 0 (0.00%) |
|  | 4 | 12 (15.58%) | 26 (20.80%) | 4 (5.56%) | 0 (0.00%) |
|  | X | 0 (0.00%) | 0 (0.00%) | 0 (0.00%) | 9 (100.00%) |
|  | Missing | 0 (0.00%) | 0 (0.00%) | 68 (94.44%) | 0 (0.00%) |
| N Stage;  n (%) | 0 | 77 (100.0%) | 6 (4.80%) | 0 (0.00%) | 0 (0.00%) |
|  | 1 | 0 (0.00%) | 88 (70.40%) | 1 (1.39%) | 0 (0.00%) |
|  | 2 | 0 (0.00%) | 31 (24.80%) | 2 (2.78%) | 0 (0.00%) |
|  | X | 0 (0.00%) | 0 (0.00%) | 1 (1.39%) | 9 (100.00%) |
|  | Missing | 0 (0.00%) | 0 (0.00%) | 68 (94.44%) | 0 (0.00%) |
| Grade;  n (%) | Well differentiated | 0 (0.00%) | 1 (0.80%) | 2 (2.78%) | 0 (0.00%) |
|  | Moderately differentiated | 38 (49.35%) | 48 (38.40%) | 14 (19.44%) | 1 (11.11%) |
|  | Poorly differentiated | 27 (35.06%) | 50 (40.00%) | 28 (38.89%) | 3 (33.33%) |
|  | Missing | 12 (15.58%) | 26 (20.80%) | 28 (38.89%) | 5 (55.56%) |
| Therapy: | | | | | |
| Neoadjuvant chemotherapy; n (%) | | 33 (42.86%) | 73 (58.40%) | 3 (4.17%) | 0 (0.00%) |
| Neoadjuvant radiotherapy; n (%) | | 44 (57.14%) | 88 (70.40%) | 1 (1.39%) | 0 (0.00%) |
| Surgery; n (%) | | 62 (80.52%) | 105 (84.00%) | 14 (19.44%) | 0 (0.00%) |
| Adjuvant chemotherapy; n (%) | | 37 (48.05%) | 70 (56.00%) | 8 (11.11%) | 0 (0.00%) |
| Adjuvant radiotherapy; n (%) | | 3 (3.90%) | 8 (6.40%) | 3 (4.17%) | 0 (0.00%) |
| Palliative chemotherapy; n (%) | | 0 (0.00%) | 2 (1.60%) | 36 (50.00%) | 0 (0.00%) |
| Palliative radiotherapy; n (%) | | 9 (11.69%) | 10 (8.00%) | 9 (12.50%) | 1 (11.11%) |
| No therapy; n (%) | | 3 (3.90%) | 6 (4.80%) | 19 (26.39%) | 7 (77.78%) |
| Missing; n (%) | | 2 (2.60%) | 0 (0.00%) | 2 (2.78%) | 1 (11.11%) |
| Liver metastases: | | | | | |
| Synchronous; n (%) | | - | - | 42 (58.33%) | - |
| Metachronous; n (%) | | 6 (7.79%) | 15 (12.00%) | 5 (16.67%) * | 0 (0.00%) |
| All-sites metastases: | | | | | |
| Synchronous; n (%) | | - | - | 72 (100.00%) | - |
| Metachronous; n (%) | | 24 (31.17 %) | 37 (29.60%) | - | 0 (0.00%) |
| Overall deaths; n (%)  Other causes; n (%) ** | | 32 (41.56%)  9 (28.13%) | 49 (39.20%)  11 (22.45%) | 64 (88.89%)  6 (9.38%); | 7 (77.78%)  2 (28.57%) |
| Steatosis at baseline; n (%)  Mild-to-moderate; n (%)  Severe; n (%) | | 21 (27.27%)  15 (19.48%)  6 (7.79%) | 39 (31.20%)  31 (24.80%)  8 (6.40%) | 27 (37.50%)  22 (30.56%)  5 (6.94%) | 3 (33.3%)  3 (33.3%)  0 (0.00%) |

Stage XM0 was considered for patients with unknown local staging but exclusion of distant metastases with CT scan. TNM stage refers to clinical staging. IQR: Interquartile range. *the percentage was calculated on a total of 30 patients, after excluding patients with baseline liver metastases. ** missing values were 45, 76, 8, and 2 in stage II, III, IV, and XM0, respectively.

Supplementary Table S2: Follow-up and treatment characteristics in patients with and without liver steatosis.

| **Table S2** | | **No Liver Steatosis**  **(n= 193; 68.2%)** | **Liver Steatosis**  **(n= 90; 31.8%)** |
| --- | --- | --- | --- |
| Overall survival follow-up duration; mean (SD) (months) | | 43.11 (30.34) | 48.97 (35.31) |
| Liver metastases follow-up duration; mean (SD) (months)  (patients without synchronous liver metastases) | | 36.49 (27.64) | 37.70 (27.80) |
| Neoadjuvant chemotherapy; n (%) | | 70 (36.3) | 39 (43.3) |
| Neoadjuvant radiotherapy; n (%) | | 88 (45.6) | 45 (50.0) |
| Surgery; n (%) | | 119 (61.7) | 62 (68.9) |
| Adjuvant chemotherapy; n (%) | | 74 (38.3) | 41 (45.6) |
| Adjuvant radiotherapy; n (%) | | 8 (4.1) | 6 (6.7) |
| Palliative chemotherapy; n (%) | | 24 (12.4) | 14 (15.6) |
| Palliative radiotherapy; n (%) | | 24 (12.4) | 5 (5.6) |
| No therapy; n (%) | | 25 (13.0) | 10 (11.1) |
| Missing; n (%) | | 4 (2.1) | 1 (1.1) |
| All-sites metastases; n (%) | Synchronous | 45 (23.3) | 27 (30.0) |
|  | Metachronous* | 43 (29.1) | 18 (28.6) |

SD, standard deviation. * percentages were calculated after excluding stage IV patients (45 in no liver steatosis group and 27 in liver steatosis group).

Supplementary Table S3: Baseline characteristics of patients with moderate/severe steatosis.

| **Table S3** | | **Moderate/Severe Liver Steatosis**  **(n= 19)** | | | |
| --- | --- | --- | --- | --- | --- |
|  | | **Overall** | **Synchronous liver metastases**  **(n=2)** | **Metachronous liver metastases**  **(n=2)** | **Deaths**  **(n=9)** |
| Age; mean (SD) | | 64.7 (15.5) |  |  |  |
| Sex;  n (%) | Male | 12 (63.2) | 1 | 1 | 5 |
|  | Female | 7 (36.8) | 1 | 1 | 4 |
| Stage;  n (%) | II | 6 (31.6) | - | - | - |
|  | III | 8 (42.1) | - | 1 | 5 |
|  | IV | 5 (26.3) | 2 | 1 | 4 |
|  | XM0 | - | - | - | - |
| Grade;  n (%) | Well | - | - | - | - |
|  | Moderately | 11 (57.9) | 2 | 1 | 3 |
|  | Poorly | 5 (26.3) | - | - | 4 |
|  | Missing | 3 (15.8) | - | 1 | 2 |

TNM stage refers to clinical staging. SD: Standard deviation.

Supplementary Table S4: CT characteristics and liver function test changes after neoadjuvant chemotherapy, defining the presence or absence of post-chemotherapy liver damage.

| **Table S4** | **Patients without post-chemotherapy liver damage**  **(n= 38, 61.3%)** | **Patients with post-chemotherapy liver damage**  **(n= 24, 38.7%)** |
| --- | --- | --- |
| Liver steatosis:  appearance  Missing | 0 (0.0%)  33 (86.8%) | 5 (20.1%)  14 (58.3%) |
| Liver volume:  Increase >10% Decrease >10%  Missing | 0 (0.00%)  0 (0.00%)  24 (58.5%) | 8 (33.3%)  3 (12.5%)  9 (37.5%) |
| AST:  Increase > 40 and doubled  Missing | 0 (0.00%)  2 (5.3%) | 8 (33.3%)  2 (8.3%) |
| ALT:  Increase > 49 and doubled  Missing | 0 (0.0%)  1 (2.3%) | 8 (33.3%)  2 (8.3%) |
| GGT:  Increase > 73 and doubled  Missing | 0 (0.0%)  8 (21.0%) | 3 (12.5%)  4 (16.7%) |

Supplementary Table S5: Follow-up, treatment, and outcome measures in patients with and without post-chemotherapy liver damage.

| **Table S5** | **Patients without post-chemotherapy liver damage**  **(n= 38, 61.3%)** | **Patients with post-chemotherapy liver damage**  **(n= 24, 38.7%)** |
| --- | --- | --- |
| Follow-up overall survival (months)  Median (IQR) | 59.5 (38-84) | 37 (26-47) |
| Follow-up liver metastases (months)  Median (IQR) | 50 (28.5-62) | 32 (19-46) |
| Adjuvant therapy | 30 (78.9%) | 18 (75%) |
| Deaths during follow-up | 5 (13.2%) | 10 (41.7%) |
| Liver metastases during follow-up | 2 (5.3%) | 5 (20.1%) |
| All sites metastases during follow-up | 14 (36.8%) | 10 (41.7%) |

IQR: Interquartile range

Supplementary Table S6: Demographic and clinical characteristics of patients with and without post-chemotherapy-induced liver damage, reported also in patients with events.

|  | | **Patients without post-chemotherapy liver damage**  **(n= 38, 61.3%)** | | | **Patients with post-chemotherapy liver damage**  **(n= 24, 38.7%)** | | |
| --- | --- | --- | --- | --- | --- | --- | --- |
|  | | **Overall** | **Metachronous liver metastases**  **(n=2)** | **Deaths**  **(n=5)** | **Overall** | **Metachronous liver metastases**  **(n=5)** | **Deaths**  **(n=10)** |
| Age at diagnosis; mean (SD) | | 59.1 (12.9) |  |  | 60.7 (12.3) |  |  |
| Sex;  n (%) | Male | 19 (50%) | 2 | 4 | 11 (45.8%) | 1 | 5 |
|  | Female | 19 (50%) | 0 | 1 | 13 (54.2%) | 4 | 5 |
| Stage;  n (%) | II | 12 (31.6%) | 1 | 3 | 8 (33.3%) | 1 | 4 |
|  | III | 26 (68.4%) | 1 | 2 | 16 (66.7%) | 4 | 6 |
| Grade;  n (%) | Well | 0 (0.0%) | 0 | 0 | 0 (0.0%) | 0 | 0 |
|  | Moderately | 19 (50.0%) | 1 | 3 | 10 (41.7%) | 1 | 4 |
|  | Poorly | 8 (21.1%) | 1 | 1 | 8 (33.3%) | 2 | 4 |
|  | Missing | 11 (28.9%) | 0 | 1 | 6 (25.0%) | 2 | 2 |

TNM stage refers to clinical staging. SD: Standard deviation.
